# Paediatricians’ knowledge, perceptions, preparedness and involvement towards paediatric antimicrobial stewardship in Pakistan: findings and the implications

**DOI:** 10.1093/jacamr/dlae193

**Published:** 2024-12-09

**Authors:** Zia Ul Mustafa, Amer Hayat Khan, Muhammad Salman, Sabariah Noor Harun, Johanna C Meyer, Brian Godman

**Affiliations:** Discipline of Clinical Pharmacy, School of Pharmaceutical Sciences, Universiti Sains Malaysia, Gelugor 11800, Penang, Malaysia; Department of Pharmacy Services, District Headquarter (DHQ) Hospital, Pakpattan 57400, Pakistan; Discipline of Clinical Pharmacy, School of Pharmaceutical Sciences, Universiti Sains Malaysia, Gelugor 11800, Penang, Malaysia; Institute of Pharmacy, Faculty of Pharmaceutical and Allied Health Sciences, Lahore College for Women University, Lahore 54000, Pakistan; Discipline of Clinical Pharmacy, School of Pharmaceutical Sciences, Universiti Sains Malaysia, Gelugor 11800, Penang, Malaysia; Department of Public Health Pharmacy and Management, School of Pharmacy, Sefako Makgatho Health Sciences University, Pretoria 0208, South Africa; South African Vaccination and Immunisation Centre, Sefako Makgatho Health Sciences University, Pretoria 0208, South Africa; Department of Public Health Pharmacy and Management, School of Pharmacy, Sefako Makgatho Health Sciences University, Pretoria 0208, South Africa; Strathclyde Institute of Pharmacy and Biomedical Science (SIPBS), University of Strathclyde, Glasgow G4 0RE, UK

## Abstract

**Introduction:**

Antibiotics are frequently prescribed for neonates and children. However, this can be excessive with inappropriate prescribing leading to increased antimicrobial resistance (AMR). Paediatricians are key initiators of antibiotics. Consequently, their awareness, perceptions, readiness and potential barriers towards hospital-based antimicrobial stewardship programmes are of considerable importance, especially in Pakistan with high rates of AMR.

**Materials and methods:**

A web-based cross-sectional survey among paediatricians from June to August 2023 using a validated questionnaire. Paediatricians from all four Provinces and the capital territory of Pakistan were invited from randomly selected public and private sector hospitals.

**Results:**

383 paediatricians participated (79.8% response rate). Most were male (87.7%), aged 35 years or less (55.4%), working in tertiary care hospitals (68.4%) and undertaking 51–100 child consultations every day (45%). Only 15% reported obtaining training on antibiotic usage, AMR and/or antimicrobial stewardship. Only 7.6% confirmed functional antimicrobial stewardship programmes in their institutions. Most had adequate knowledge of antibiotic use and AMR. However, key issues were not fully understood with only 27.4% believing antibiotics were being overused among children. Paediatricians with less experience, and who undertook fewer consultations per day, had significantly lower knowledge scores. Most participants were prepared to initiate antimicrobial stewardship programmes; however, perceived barriers included a lack of online learning sources, treatment guidelines and support from hospital administration.

**Discussion:**

Paediatricians had appropriate knowledge about antibiotic use and AMR although concerns with antibiotic use. Important barriers to integrating antimicrobial stewardship programmes were identified, which need addressing for these to become routine.

## Introduction

Antibiotics are one of the most frequently prescribed medicines among neonates and children.^1,2^ However, over time, antibiotics have become less effective through increasing antimicrobial resistance (AMR) arising from their overuse and misuse.^3,4^ AMR is currently one of the greatest threats to global health. It was estimated that in 2019, AMR accounted for 1.27 million deaths, with the highest mortality in sub-Saharan African and South-Asian countries.^5^ There are also appreciable morbidity and economic consequences associated with AMR, which is highest among low- and middle-income countries (LMICs).^6–9^

Neonates and children are at particular risk of AMR including from resistance attributable to neonatal sepsis, which accounted for an estimated 25 692 deaths in 2015 (16 486 to 39 660).^10^ When this risk is combined with higher mortality rates, generally from infectious diseases in neonates and children, there is an increasing need to carefully manage this age group.^11,12^ Overall, 20%–30% of paediatric patients are reported to have multi-drug resistant organisms, with higher rates of 66%–90% in Africa and the Middle East.^13–15^ In Pakistan, the data concerning AMR, its economic consequences, and mortality are scarce;^16^ however, a few studies have revealed high rates of AMR in children.^17–20^

The excessive and inappropriate use of antibiotics are one of the principal factors associated with AMR.^14,21^ A cohort study conducted among eight LMICs, including Pakistan, revealed 4.7 antibiotic episodes per child in a year.^22^ Other studies in Pakistan have also documented considerable prescribing and dispensing of antibiotics among neonates and children. Excessive prescribing of antibiotics has been observed in up to 100% of children with upper respiratory tract infections (URTIs) treated by physicians in ambulatory care clinics in Pakistan, including hospital outpatients.^23–27^ These high inappropriate rates of antibiotic prescribing have been exacerbated by requests from parents.^23,28^

Alongside ongoing concerns with inappropriate prescribing of antibiotics for children, there are also concerns with current extensive dispensing of antibiotics without a prescription in Pakistan.^25,29^ This includes antibiotics for self-limiting conditions such as URTIs and acute diarrhoea.^22,29,30^ In addition, appreciable dispensing of antibiotics from the World Health Organization’s’ (WHO) ‘Watch’ list of antibiotics with their greater resistance potential.^29^ There has also been extensive inappropriate prescribing of antibiotics among neonates and children in both secondary and tertiary hospitals in Pakistan, with up to 97% of hospitalized children being prescribed antibiotics.^31–35^ This includes an increase in the prescribing of antibiotics from the WHO Watch list as part of the AWaRe (Access, Watch and Reserve) classification, which is similar to other LMICs.^31,34,36^ Antibiotics from the Watch group, including azithromycin and a number of the cephalosporins and quinolones, should ideally only be prescribed in critical conditions due to their greater chance of resistance development.^37^ Antibiotics in the ‘Reserve’ category, which include fifth-generation cephalosporins and some carbapenems, should only prescribed in multi-drug resistance cases.^37,38^ This is important with the United Nations General Assembly (UN GA) on AMR recommending that ‘Access’ antibiotics should now constitute a minimum of 70% of antibiotics prescribed and dispensed to reach AMR mortality goals.^39,40^ This is up from a previous WHO recommendation of 60% of antibiotics used in a sector being from the Access group.^36–38^

The COVID-19 pandemic further increased antibiotic utilization in Pakistan including Watch antibiotics, despite limited evidence of bacterial co-infections or secondary infections in these patients.^41–45^ This increase in prescribing of antibiotics from the Watch list has further enhanced AMR in Pakistan, considering their increased potential for resistance.^4,36,46^

In view of increasing AMR rates, particularly in LMICs, international organizations such as the WHO initiated a number of activities to reduce AMR,^7,47^ including the WHO Global Action Plan (GAP).^48^ In line with the recommendations of the WHO, the Government of Pakistan formulated its ‘National Action Plan (NAP)’ against AMR, which emphasizes appropriate antibiotic use to reduce AMR.^49,50^ However, there have been issues and challenges with the implementation of Pakistan’s NAP.^51^ A key element of NAPs is the encouragement of antimicrobial stewardship activities to improve future antibiotic use given global concerns.^51,52^ This includes the implementation and integration of antimicrobial stewardship programmes into prescribing activities where these have not taken place, or where antimicrobial stewardship activities have been limited. To date, hospital-based antimicrobial stewardship programmes have been developed and implemented in many countries to improve antibiotic prescribing and reduce AMR.^52–56^ However, there have been concerns regarding available resources and personnel to effectively implement and undertake antimicrobial stewardship programmes in LMICs.^57^ This is changing, however, with an increasing number of antimicrobial stewardship programmes now being implemented across multiple LMICs.^55,56,58–61^ Alongside this, there are also an increasing number of online educational initiatives, including accreditation programmes, to help with the implementation of antimicrobial stewardship programmes and subsequently reduce the development of AMR.^62–64^

Currently, >40% of the population in Pakistan are below 18 years of age.^65^ Consequently, in view of currently high rates of inappropriate prescribing of antibiotics among this population group in Pakistan, implementation of antimicrobial stewardship programmes is critical to improve future antibiotic utilization.^24,31–35^ As a result, reduce rising rates of AMR in the country.^16,19,50^ This is particularly important given the current high levels of inappropriate prescribing of antibiotics in Pakistan, fuelled by the recent COVID-19 pandemic, and concerns that these high rates will continue unless addressed.^42–44,46^

Previous studies have shown insufficient awareness, training and implementation of antimicrobial stewardship programmes among healthcare workers (HCWs) in general in Pakistan.^18,28,66–70^ Having said this, we are not aware of any study that has been undertaken specifically among paediatricians in Pakistan, regarding their perceptions, preparedness, involvement and barriers with implementing antimicrobial stewardship programmes among neonates and children admitted to their hospitals. Consequently, we sought to address this important evidence gap, given the continuing concerns with current prescribing practices of antibiotics in this vulnerable population.^29–35^ Subsequently, we will seek to use the findings to provide future guidance to all key stakeholder groups in Pakistan who are involved in managing this priority group.

## Materials and methods

### Study design, location and population

We conducted a cross-sectional survey using a web-based questionnaire. The target population included paediatricians from all four provinces and the capital territory of Pakistan, working in both the public and private sectors, and in secondary and tertiary care hospitals, but not currently practising in primary care settings. We targeted hospitals for this study as these are the health facilities in Pakistan where sick children are typically treated. We also concentrated on paediatricians as they are a key group of healthcare professionals (HCPs) in Pakistan, influencing non-paediatricians with respect to the prescribing of antibiotics in neonates and children. Consequently, if there are concerns with antibiotic use and antimicrobial stewardship initiatives among paediatricians, these kind of issues and concerns are likely to be greater among other HCPs. Paediatricians in Pakistan currently only work in hospitals and not in primary healthcare centres (PHCs); however, their influence is much wider. This includes physicians involved with treating children in PHCs, who are influenced by local paediatricians. This scenario is typical of several LMICs where nurses and other HCWs generally treat patients in PHCs under the guidance of other clinicians.^71–73^

In Pakistan, the Department of Health of the Punjab Government has two divisions. First, the Specialized Health and Medical Education Department, which is the controlling authority of tertiary/teaching hospitals, and mainly established in metropolitan cities of the province, serving referral hospitals. The second division is the Primary and Secondary Healthcare Department, which comprises secondary hospitals, where sick neonates and children are typically treated. This incorporates district headquarters, tehsil headquarters, ambulatory care health settings including rural health centres and basic health units.

### Data collection tool

We used a previously validated data collection tool for this study.^70,74–77^ The reliability or internal consistency of the study instrument was assessed using Cronbach’s Alpha, obtaining a value of >0.7. In addition, a pilot study was conducted among 20 paediatricians before initiation of the main study. All pilot study participants stated that they fully understood the contents of the questionnaire. However, minor amendments were suggested, which were subsequently incorporated into the final study instrument. The final questionnaire was made available as a survey, using Google Forms, and consisted of the following sections:

- Section I contained questions about the demographic characteristics of the respondents, including any training/research related to antimicrobial stewardship programmes and the current status of antimicrobial stewardship programmes in their hospital.
- Section II consisted of 10 questions to evaluate knowledge about antibiotic use and AMR. Each question had three options, namely ‘Yes’ ‘No’ and ‘Don’t know’. Each correct answer was scored as 1, while the other two responses, i.e. an incorrect answer and ‘Don’t know’, were scored as zero. A total knowledge score was calculated for each respondent with a score >7 considered as good, <5–7 as moderate and <5 as poor, similar to previous studies.^70,75–77^
- Section III comprised 10 statements to evaluate respondents’ perceptions regarding the potential causes of AMR in their hospitals. Respondents were requested to select a suitable option from a 5-point Likert scale.^78^ A total score was computed by adding the scores of all 10 items, with higher scores indicating better perceptions regarding the potential causes of AMR.
- Section IV evaluated respondents’ perceptions about antibiotic use and AMR. In this section, nine statements were provided with again a 5-point Likert response scale. A total score (up to a maximum of 45) was computed by adding the scores of all items. Scores >33 were considered as good scores, <32–22 as moderate and <22 as poor scores, again similar to previous studies.^70,75–77^
- Section V contained a list of different sources of information on appropriate antibiotic use.
- Section VI dealt with respondents’ preparedness towards implementing antimicrobial stewardship programmes in their hospitals.
- Section VII consisted of 10 statements about possible to ways to reduce AMR and subsequently implement antimicrobial stewardship programmes in respondents’ hospitals.
- Section VIII contained 11 statements about possible barriers with the implementation of antimicrobial stewardship programmes in hospitals. Respondents could select any number of appropriate statements indicating ‘Yes’ or ‘No’ as applicable to their hospitals. The same validated scoring metrics were used, as used in previous studies.^70,75–77^

### Sample size determination

At the time of the study, the total number of paediatricians working in Pakistan was unknown. Consequently, we contacted a representative from the Pakistan Pediatric Association to collect this information. According to their estimates, nearly 5000 paediatricians were practising in Pakistan at that time, including across all hospital types and sectors. A sample size calculation was performed using Raosoft online sample size calculator 206-525-4025 (USA). Assuming a total paediatrician population of 5000, with an expected response distribution of 50%, a confidence interval of 95% and a margin of error of 5%, the minimum calculated sample required for the current study was 357. However, we distributed questionnaires to 480 participants in secondary and tertiary hospitals to account for paediatricians not responding.

### Data collection procedures and analysis

Overall, in Pakistan, there are ∼1200 public sector hospitals, principally secondary care hospitals,^79^ with just over 500 private hospitals operational in the country. Out of these, 21 tertiary care hospitals and 36 secondary care hospitals were randomly selected throughout the country for this study, principally targeting hospitals where paediatricians will be most likely practising. Most of the hospitals targeted for the study were in Punjab Province since currently >70% of paediatricians in Pakistan work in Punjab. In other provinces, at least one tertiary care hospital and two secondary care hospitals were targeted for the study. For instance, at the time of the study in Sindh and KPK, ∼900 and 600 paediatricians, respectively, were working in these two provinces, with ∼10% of the paediatricians in these provinces targeted for the study. By using this approach, we sought to limit any bias in our findings by including both public and private sector secondary and tertiary hospitals, and subsequently targeting paediatricians based on the anticipated number of paediatricians working in that province at the time of the study. Following this, all potential participants in the targeted hospitals were contacted through email and WhatsApp with the assistance of the Pakistan Pediatric Association and personal contacts. This translated to typically 10–15 paediatricians per targeted tertiary hospital (both public and private sectors) alongside 5–8 paediatricians per secondary care hospital (both public and private). Participants were contacted between June and August 2023. A link to the online version of the questionnaire was shared through email and WhatsApp, with the support of institutional societies and the administrative departments of the relevant institutions. To increase the response rate, a gentle reminder was sent to all potential respondents after 1 week.

### Statistical analysis

We used SPSS version 22 for performing the statistical analysis. Frequencies and percentages were calculated for categorical variables, with median (quartiles) for continuous variables. To summarize ordinal data and facilitate interpretation, responses to the 5-point Likert scale items, i.e. ‘Strongly agree’ and ‘Agree’ were collapsed into one category, while ‘Disagree’ and ‘Strongly disagree’ were collapsed into one category. Similarly, for section VI, ‘Very poor’ and ‘Poor’ were collapsed into one category, and ‘Good’ and ‘Very good’ also collapsed into one category. The association of demographic variables with outcome variables (knowledge and perception scores), e.g. awareness of antibiotic use and AMR, causes of AMR, perceptions of AMR, preparedness towards antimicrobial stewardship programmes, and approaches to combat AMR, were assessed using the Mann–Whitney *U*-test and/or Kruskal–Wallis Test, where appropriate. *P* values <0.05 (two-sided) were considered statistically significant.

### Ethical considerations

Ethical approval for the current study was obtained from the Office of Research, Innovation and Commercialization, Lahore College for Women University, Lahore, Pakistan, and from the ethics committees/office of the administrators of the participating hospitals. Informed consent was obtained from all respondents before participation by requesting them to click on an ‘Agree’ button on the online platform before proceeding with completing the questionnaire. This step was mandatory for their active participation. Furthermore, no personal information was recorded. Data was subsequently coded and stored in a password protected Microsoft Excel^®^ sheet, which was accessible only to the researchers.

## Results

### Demographic characteristics of study participants

Out of the 480 questionnaires distributed via email, 383 paediatricians replied, translating into a response rate of 79.8%. Table S1 (available as Supplementary data at *JAC-AMR* Online) (Supplementary material) provides further detail on the number of hospitals contacted in each province and the total number of paediatricians who replied, e.g. in Punjab nearly 3000 paediatricians were working in the province at the time, with ∼10% contacted for the study. Response distributions were similar in the other provinces. Participants’ characteristics are summarized in Table 1. There was a preponderance of male paediatricians and those aged 35 years or younger, while 43.1% had 6 to 10 years professional experience. Most paediatricians were providing medical care at tertiary healthcare settings throughout Pakistan (68.4%) and 45.2% reported providing consultations to between 51 and 100 children per day. Only 15.1% of the study participants reported obtaining training on antibiotic use, AMR and/or antimicrobial stewardship in the past year. Furthermore, only 7.6% stated that their institution had a functional antimicrobial stewardship programme at the time of completing the questionnaire.

**Table 1.** Demographic details of the sample

Variable	Number (%)
Age (years)	
≤35	212 (55.4)
36–45	122 (31.9)
>45	49 (12.8)
Sex	
Male	336 (87.7)
Female	47 (12.3)
Experience (years)	
≤5	157 (41.0)
6–10	165 (43.1)
>10	61 (15.9)
Healthcare institute	
Secondary care	121 (31.6)
Tertiary care	262 (68.4)
Number of paediatric consultations per day	
≤25	20 (5.2)
26–50	77 (20.1)
51–100	173 (45.2)
>100	113 (29.5)
Locality	
Rural	38 (9.9)
Urban	345 (90.1)
Training on antibiotics use/antibiotic resistance/antimicrobial stewardship in previous year	
Yes	58 (15.1)
No	325 (84.9)
Institution/department has a functional paediatric antimicrobial stewardship programme	
Yes	29 (7.6)
No	354 (92.4)

### Paediatricians’ awareness about antibiotic use and antibiotic resistance

An appreciable number of paediatricians knew antibiotics were not usefulness in treating viral infections (96.6%) and 83.3% also knew these agents can cause secondary infections by destroying the body’s normal flora (Table 2). All participants were aware that antibiotics can cause allergic reactions. Only 65.8% of surveyed paediatricians knew that skipping antibiotic doses can contribute to AMR, and only 62.9% provided correct responses to the question assessing knowledge of antibiotic cross-resistance. While all the study participants reported being aware of AMR, only 41.3% reported that they were taught about AMR in their undergraduate or postgraduate curricula. Moreover, 25.3% stated that they were aware of antimicrobial stewardship; however, only 11.2% reported they were taught about antimicrobial stewardship in their curriculum (Table 2). The median (IQR) knowledge score within this domain was 7 (6–8).

**Table 2.** Respondents’ awareness about antibiotics and antibiotic resistance

Statements/questions	Number (%)		
	Correct/yes	Incorrect/no	Don’t know
Antibiotics can be useful in treating viral infections	370 (96.6)	13 (3.4)	—
Antibiotics can cause secondary infections by killing normal flora	319 (83.3)	23 (6.0)	41 (10.7)
Antibiotics can cause allergic reactions	383 (100)	—	—
A resistant bacterium cannot spread in healthcare institutions	269 (70.2)	32 (8.4)	82 (21.4)
Skipping one or two doses does not contribute to the development of antibiotic resistance	252 (65.8)	76 (19.8)	55 (14.4)
Cross-resistance is the condition in which the resistance occurs to a particular antibiotic that often results in resistance to other antibiotics, usually from a similar class	241 (62.9)	35 (9.2)	107 (27.9)
Pain and inflammation without any possibility of infection are indications for antimicrobial therapy?	321 (83.8)	51 (13.3)	11 (2.9)
Are you aware of antibiotic resistance?	383 (100.0)	—	—
Have you been taught antibiotic resistance in your curriculum?	158 (41.3)	197 (51.4)	28 (7.3)
Are you aware of antibiotic stewardship?	97 (25.3)	276 (72.1)	10 (2.6)
Have you been taught antibiotic stewardship in your curriculum?	43 (11.2)	328 (85.6)	12 (3.1)

### Potential causes for antibiotic resistance according to paediatricians

**Figure 1.**
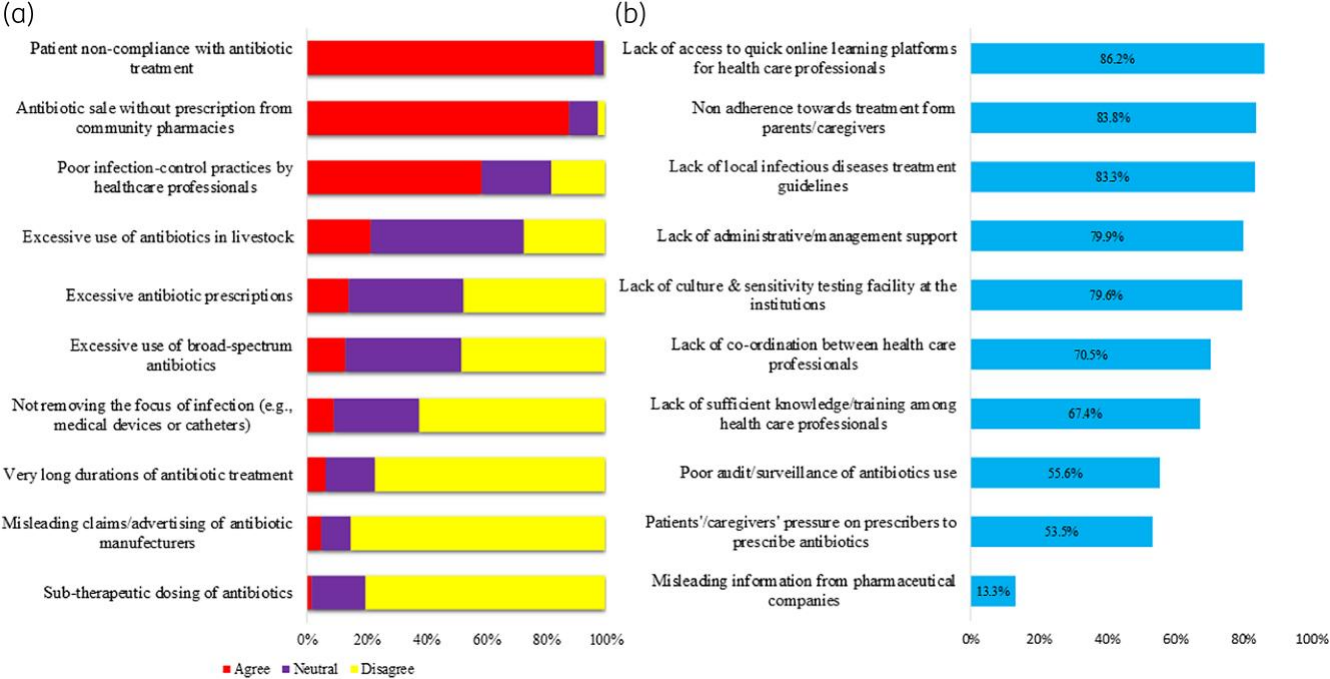
Perceived causes of AMR (a) and barriers to implementing antimicrobial stewardship programmes (b).

Paediatricians perceived the top three causes of AMR were: (i) patients’ non-compliance with treatment, (ii) the availability of antibiotics without a prescription and (iii) poor infection-control practices by HCWs (Figure 1a). However, a relatively small percentage agreed that excessive use of antibiotics, excessive use of broad-spectrum antimicrobials and a longer duration of antibiotic treatment were all linked with increasing AMR. Overall, the median (IQR) knowledge score within this domain was 28 (25–30).

### Paediatricians’ perceptions about antibiotic use and antibiotic resistance

**Figure 2.**
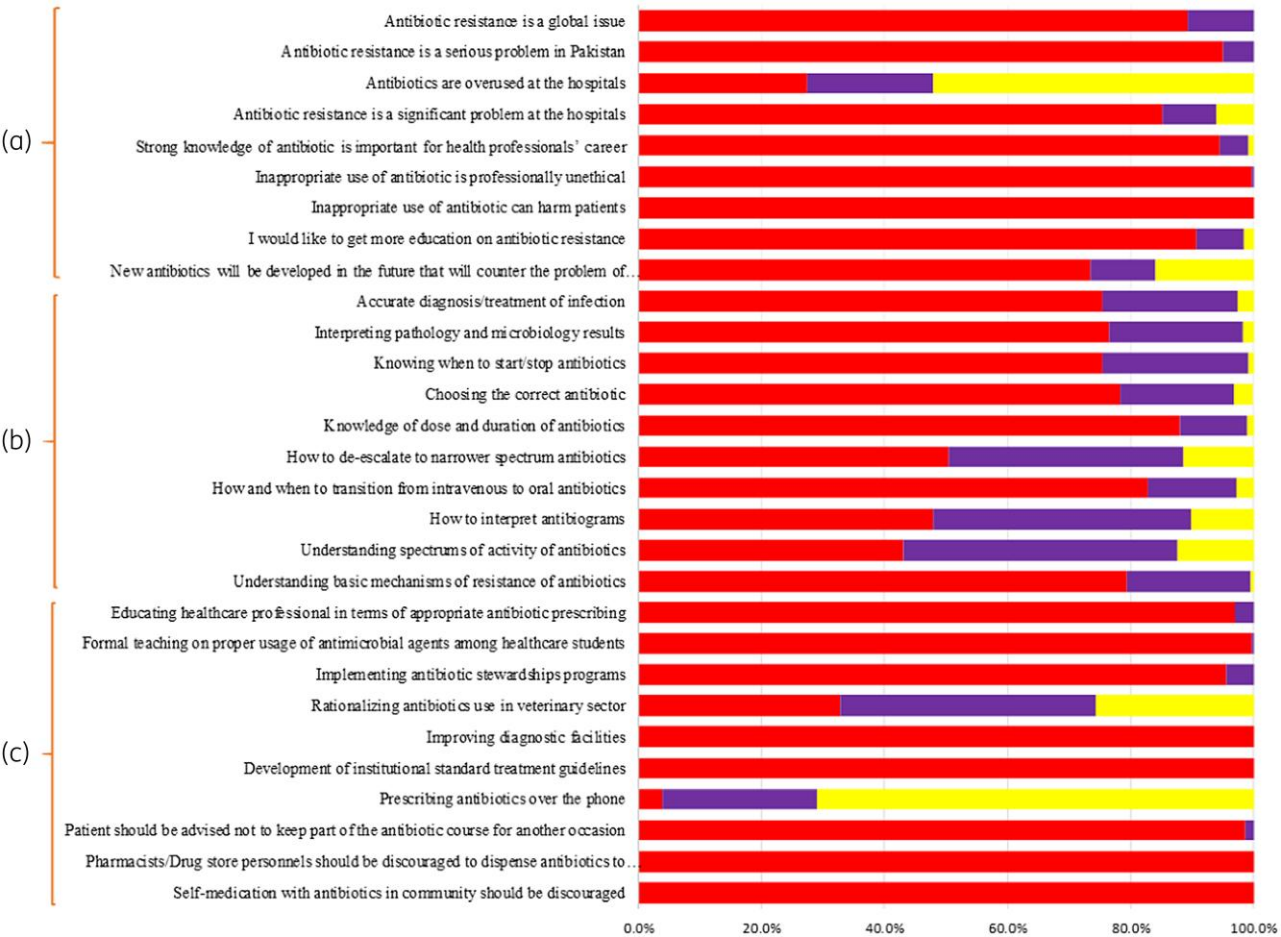
Respondents’ perceptions towards antibiotic use and antibiotic resistance (a), preparedness for antimicrobial stewardship (b) and ways to reduce antibiotic resistance (c). The colour code is the same as Figure 1.

Most study participants believed AMR is a global issue, 95.0% perceived AMR as a serious concern in Pakistan and 85.1% that AMR is a significant problem in their hospital (Figure 2a). Despite this, only 27.4% thought antibiotics were being overused, and 73.1% that new antibiotics will soon be made available to counteract AMR. Encouragingly, most paediatricians showed willingness to learn more about AMR (Figure 2a). The median (IQR) score within this domain was 39 (36–42).

### Resources used for learning about antibiotic use and antibiotic resistance

The top three most commonly employed sources to stay up-to-date with current antibiotic recommendations were textbooks or study guides, infectious disease specialists or seniors, and fellow doctors/colleagues (Figure S1).

### Paediatricians’ perceptions of preparedness in antimicrobial stewardship

Participants’ perceptions of their preparedness to implement antimicrobial stewardship programmes are shown in Figure 2b. Overall, study participants felt they were quite good at accurately diagnosing and providing treatment, interpreting laboratory data and understanding the basic mechanisms of antibiotic resistance. Participants also felt they were quite good at knowing when to start or end antibiotic treatment, choosing the right antimicrobial, as well as knowing how to de-escalate antibiotics and switch from IV to oral antibiotic therapy. There was, however, considerable variation with interpreting antibiograms. The median (IQR) score within this domain was found to be 45 (43–47).

### Ways to reduce antibiotic resistance according to paediatricians

As shown in Figure 2c, study participants believed the principal strategies to tackle AMR are to develop standard treatment guidelines, curb prescribing of antibiotics without a prescription, discourage self-medication with antibiotics and improving diagnostic facilities.

### Perceived barriers towards implementing antimicrobial stewardship programmes

Perceived barriers to implementing antimicrobial stewardship programmes among surveyed paediatricians included lack of access to quick online learning programmes/courses and patients’ non-adherence to prescribed medication (Figure 1b).

Female physicians had significantly higher scores related to appropriate prescribing of antibiotics and AMR; however, they had lower scores regarding their preparedness towards implementing antimicrobial stewardship programmes (Table 3). Paediatricians with fewer years of medical experience had significantly lower knowledge scores regarding antibiotics and AMR, compared to those with a longer duration of experience. Physicians who were providing consultations to a larger number of paediatric patients had better knowledge scores regarding antibiotics and reported better preparedness towards implementing antimicrobial stewardship programmes. Encouragingly, those who reported having received training regarding antimicrobial stewardship programmes were more prepared to implement these programmes (*P* = 0.008).

**Table 3.** Association of demographic variables and knowledge of antibiotics, AMR and antimicrobial stewardship

Variables	General knowledge of antibiotics and AMR		Causes of AMR		Perception of AMR		Preparedness towards antimicrobial stewardship		Approaches to tackle AMR	
	Mean rank	*P* value	Mean rank	*P* value	Mean rank	*P* value	Mean rank	*P* value	Mean rank	*P* value
Age (years)		0.056		0.299		0.667		0.872		0.658
≤35	180.26		199.81		196.52		189.73		189.58	
36–45	209.08		183.33		186.84		199.15		199.15	
>45	200.27		179.80		185.27		184.66		184.66	
Sex		**0**.**011**^a^		**0**.**034**		0.424		**0**.**002**		0.240
Male	186.70		187.54		193.88		185.55		194.47	
Female	229.87		223.85		179.98		238.11		174.36	
Experience (years)		**<0.001**		0.527		0.183		0.774		0.884
≤5	163.04		199.17		202.85		188.07		189.25	
6–10	214.55		188.80		188.47		196.60		192.66	
>10	205.57		182.22		173.61		189.67		197.31	
Healthcare institute		0.132		0.089		0.504		**0**.**049**		0.113
Secondary care	204.29		177.90		186.47		208.35		178.88	
Tertiary care	186.32		198.51		198.51		184.45		198.06	
Paediatric consultations/day		**<0.001**		0.529		0.364		**<0.001**		0.064
≤25	145.98		177.60		163.15		139.40		224.83	
26–50	170.69		179.07		205.39		183.51		210.68	
51–100	181.24		193.41		186.28		176.55		192.11	
>100	231.14		201.20		196.73		230.74		173.30	
Locality		0.648		0.472		0.676		0.903		0.175
Rural	199.64		204.21		184.91		194.07		214.96	
Urban	191.16		190.66		192.78		191.77		189.47	
Antimicrobial stewardship training during past year		0.117		0.123		0.190		**0**.**008**		**0**.**006**
Yes	212.58		212.56		174.56		227.13		155.73	
No	188.33		188.33		195.11		185.73		198.47	
Functional paediatric antimicrobial stewardship programme at hospital		0.062		0.605		0.642		**0**.**026**		0.968
Yes	228.17		181.81		182.86		235.98		191.22	
No	189.04		189.04		192.83		188.40		192.06	

^a^Bold font is statistically significant.

## Discussion

We believe this is the first study conducted among paediatricians in Pakistan to ascertain their knowledge, awareness, perceptions, readiness and involvement towards implementing antimicrobial stewardship programmes in their hospitals. This is important given rising rates of AMR in Pakistan as well as high levels of inappropriate prescribing of antibiotics among neonates and children, including during the recent pandemic.^27,31,34,43–46,66,80^ Concerns also include high rates of prescribing of antibiotics from the WHO Watch list.^34,43,44,66^ This situation urgently needs addressing to meet the new UN GA AMR target for Access antibiotics.^39,40^

Encouragingly, there was a high response rate of 79.8% among participating paediatricians. We believe the high response rate was facilitated by obtaining contact details from the professional society and through personal contacts. Most participating paediatricians also considered AMR a global concern, and particularly in hospitalized settings. However, they typically possessed insufficient knowledge of antibiotic use and AMR. Participating paediatricians were aware of the potential causes of AMR, including non-compliance among patients, antibiotics being readily available without a prescription and poor infection prevention and control activities among HCWs, which is similar to other LMICs.^57,60,81,82^ Paediatricians with longer work experience also showed better antibiotic use and awareness compared to their colleagues with less experience, which is similar to the findings from Nepal.^83^ Having said this, most participating paediatricians did not believe that any over prescribing of antibiotics in their hospitals was a major driver of AMR. This needs to be addressed, with antibiotic use among hospital clinicians an ongoing concern. A number of studies have demonstrated excessive prescribing of antibiotics among hospitalized neonates and children in Pakistan, which includes Watch and Reserve antibiotics.^27,31,32,34,66,80^ This includes rates up to 99% among young children and neonates in paediatric intensive care units and neonatal medical wards in tertiary hospitals in Pakistan, and 91% among children in paediatric medical wards.^32^ These rates demonstrate appreciable differences in reality between knowledge scores regarding antibiotics and AMR and paediatricians’ actual prescribing practices. This discrepancy may arise from the fact that only a limited number of participating paediatricians received education on AMR and antimicrobial stewardship programmes as part of their undergraduate and postgraduate curriculum, and had received any training on these aspects in the last year. This is similar to a study from Iran where there was also insufficient knowledge among physicians regarding antibiotic use and AMR.^84^ In addition, in Nigeria only 28.2% of physicians in leading hospitals had heard of antimicrobial stewardship.^85^ Similarly in Zambia, there was relatively limited knowledge regarding antimicrobial stewardship activities among physicians and pharmacists even in leading tertiary hospitals.^86^ Encouragingly, an Egyptian study showed that with sufficient training, prescriber practices regarding antimicrobial stewardship activities can be improved,^87^ with similar findings in other LMICs.^60,66,88,89^ Consequently, periodic training and inclusion of AMR and antimicrobial stewardship modules in the curriculum, coupled with professional development activities post-qualification, can improve paediatricians’ knowledge about the rational use of antibiotics. Promisingly, most study participants reported that they felt prepared to initiate and execute antimicrobial stewardship programmes in their hospitals, which includes addressing current barriers. This is important with only 7.6% of institutions among the study participants currently having functional groups performing antimicrobial stewardship activities, which is very different from European hospitals.^90^ However, this is an improvement over previous studies and their findings in Pakistan.^68,91^

Current barriers to implementing antimicrobial stewardship activities include concerns with de-escalating antibiotics and rapidly shifting from parenteral to oral routes as well as accurately interpreting antibiograms. These issues need to be addressed going forward with appropriate training and continuing professional development activities, especially if paediatricians are to effectively lead the introduction of appropriate antimicrobial stewardship programmes in their hospitals. Alongside this, subsequently monitor antibiotic utilization patterns. Practical activities to successfully implement appropriate antimicrobial stewardship programmes included addressing the current lack of support from hospital administrators, overcoming a lack of culture and sensitivity testing facilities even in tertiary hospitals, as well as addressing limited availability of online sources to guide treatment decisions.^31,32,34,68,92^ In addition, overcoming patients’ non-adherence to prescribed antibiotics, although this is less of an issue in the hospital environment. The current lack of online treatment guidelines should now be less of an issue following the online publication of the WHO AWaRe Book and guidance.^37,38^ The lack of hospital administrator support, including a lack of culture and sensitivity testing facilities, can also potentially be addressed by pointing out the need to appreciably improve antibiotic prescribing as part of the NAP as well as to meet the Access and AMR goals in the UN GA proclamation.^39,49,50^ However, it is important to acknowledge that considerable barriers still remain with implementing antimicrobial stewardship programmes in hospitals in Pakistan.

Potential antimicrobial stewardship activities could include the introduction and monitoring of compliance rates to infectious diseases treatment guidelines based on the AWaRe Book, including agreed quality indicator targets.^37,38,93^ This builds on the study by Shakeel *et al.* among paediatricians.^94^ Alongside this, investment in appropriate diagnostic and surveillance facilities to reduce current high rates of empiric antibiotic prescribing in hospitals.^31,34,44,66,95^ These proposed activities can build on recent educational activities across LMICs to increase the number of antimicrobial stewardship programmes.^61–63^ Yet, care is needed when instigating antimicrobial stewardship programmes in neonates and children, because children are not just little adults due to their unique body composition which changes over time. These issues must be taken into consideration when designing and implementing any antimicrobial stewardship programme in this target population.^14,96^ Pharmacokinetics and pharmacodynamics properties of medications including antibiotics are also variable in children,^97^ with a number of antibiotics causing frequent adverse drug reactions in this population compared to minimal adverse drug reactions in adults.^98^ Alongside this, usually children are not included in drug dose-determination studies, potentially leading to their off-label use. Doses of antibiotics for children may also well be determined by extrapolation from the adult dose, which can be an issue, with similar concerns regarding the optimal duration of antibiotic therapy in this population.^95,99,100^ Consequently, extra care is needed when introducing and integrating antimicrobial stewardship programmes among neonates and children. Furthermore, children’s growth patterns and dietary habits can present challenges, e.g. the chelation of fluoroquinolones with milk and formula.^101^ Finally, the long-term effects of antibacterial therapy may also result in alterations in gut microbiome, immune system and atopic diseases, affecting possible antimicrobial stewardship programmes and their monitoring.^102^

Alongside encouraging antimicrobial stewardship programmes among paediatricians as part of the NAP, the Government of Pakistan also needs to instigate activities to address current high rates of purchasing of antibiotics without a prescription.^25,29,30,103,104^ This also includes addressing appreciable dispensing of antibiotics without a prescription from the Watch and Reserve list, which is not helped by current regulations.^22,29,30,104,105^ A number of activities have now been proposed to address these concerns. This includes greater education of community pharmacists and their assistants regarding antibiotics, AMR and antimicrobial stewardship programmes, changes in the current Drug List as well as greater monitoring of dispensing practices.^29,82,104–107^ This is similar to suggestions for paediatricians.^24,31,34,35^

The Government and other key stakeholders, including paediatricians, also need to consider educational interventions among parents.^103,108^ The rationale for this approach is that if parents become more aware of the lack of effectiveness of antibiotics in essentially viral infections, and their harm if overused, they may exert less pressure on HCPs to prescribe or dispense antibiotics for self-limiting conditions such as URTIs.^103,108–110^ We will be following this up in future research projects involving parents.

We are aware of several limitations with our study. First, we recruited study participants through contacts of the professional society and co-authors; consequently, some degree of bias exists. Second, issues of coverage error and selective participation may exist from online surveys.^111^ However, there was a high response rate at the randomly selected hospitals (79.8%). We also did not ascertain the extent of any financial and administrative support as well as rewards for study participants to develop and implement antimicrobial stewardship programmes in their hospitals. Finally, we are aware that only a limited number of female paediatricians took part; however, this reflects the current situation in Pakistan where the number of female paediatricians is less compared to male paediatricians. Despite these limitations, we believe our findings will be helpful to key stakeholders in Pakistan to address the ongoing crisis of AMR among neonates and children.

### Conclusions

Most paediatricians working in public and private sectors of Pakistan possessed insufficient knowledge of antibiotic use and AMR. They were aware of the potential causes of AMR, identifying non-compliance from patients, availability of antibiotics without prescriptions and poor infection prevention and control measures among HCWs. However, most did not identify that overprescribing of antibiotics in the hospitals in Pakistan is a major driver of AMR. Overall, antibiotic prescribing among paediatricians remains a concern with high rates of prescribing, which includes antibiotics from the Watch list. Most of the study participants were prepared to initiate and execute antimicrobial stewardship activities in their hospitals including addressing current barriers. However, practical barriers remain, including the lack of online learning sources, lack of indigenous treatment guidelines and insufficient support from hospital administration. An additional measure, likely to prove helpful, is to monitor antibiotic prescribing patterns against agreed quality targets based on the recently launched AWaRe Book. Going forward, these areas need to be prioritized to urgently reduce AMR in neonates and children as part of the NAP of Pakistan.

## Supplementary Material

dlae193_Supplementary_Data

## Data Availability

Additional data are available from the authors upon reasonable request.

## Supplementary data

Figure S1 and Table S1 are available as Supplementary data at *JAC-AMR* Online.
