# Supplementary material for: Paediatricians’ knowledge, perceptions, preparedness and involvement towards paediatric antimicrobial stewardship in Pakistan: findings and the implications
Source: JAC Antimicrob Resist. 2024 Dec 9;6(6):dlae193. doi: 10.1093/jacamr/dlae193 (PMC11631149; doi:10.1093/jacamr/dlae193)
Supplement: dlae193_Supplementary_Data [file dlae193_supplementary_data.docx]

**Supplementary file: Paediatricians’ Knowledge, Perceptions, Preparedness, and Involvement towards Paediatric Antimicrobial Stewardship in Pakistan: Findings and the Implications**

**Table S1: Targeted Hospitals per Province**

| **Province** | **Public sector** | | | | **Private sector** | | | | **Total** |
| --- | --- | --- | --- | --- | --- | --- | --- | --- | --- |
|  | TH | Participants | SH | Participants | TH | Participants | SH | Participants |  |
| Punjab | 10 | 97 | 11 | 74 | 2 | 23 | 3 | 18 | 212 |
| Sindh | 2 | 19 | 5 | 34 | 1 | 8 | 1 | 6 | 67 |
| KPK | 2 | 17 | 4 | 22 | 1 | 8 | 3 | 14 | 61 |
| Balochistan | 1 | 8 | 4 | 15 | - |  | 1 | 3 | 26 |
| Capital territory | 1 | 4 | 3 | 7 | 1 | 3 | 1 | 3 | 17 |
| Total | 16 | 145 | 27 | 152 | 5 | 42 | 9 | 44 | 383 |

NB: KPK-Khyber Pakhtunkhwa; SH-secondary hospital; TH-tertiary hospital

**Figure S1: Sources of information of antibiotic use and antimicrobial resistance**
